# State of art of neuro-urology in the Moroccan context

**DOI:** 10.1080/2090598X.2021.2007465

**Published:** 2021-12-07

**Authors:** Jihad El Anzaoui, Abdelghani Ammani

**Affiliations:** Department of Urology, Military Hospital My Ismail Meknes, Sidi Mohamed Ben Abdellah University of Fes, Meknes, Morocco

**Keywords:** Neuro-urology, functional urology, fellowship, urology, continuing medical education

The insufficiency of the practice of neuro-urology and functional urology in Morocco is a general concern [1]. The rarity of published clinical and epidemiological studies preclude a precise knowledge of the epidemiological features of neuro-urological disorders in the Moroccan context. The existing epidemiological data are not efficiently informative because they often concern a single department or a single practitioner. According to Benchekroun et al. [2], neurogenic bladder represents 0.6% of all urology departments admissions. The neurological and neuro-urological disorders are known to have a heavy social impact especially from progressive loss of autonomy. These patients require both multidisciplinary medical care and close social follow-up.

In the Moroccan context, although medical care has progressed in recent years with the appearance on the Moroccan market of new therapeutic tools such as new classes of anti-cholinergic drugs (tolterodine, solifenacin) and neurostimulation devices, and the widespread use of botulinum toxin, it remains insufficient for several reasons, dominated by the limited number of urodynamic centers. This insufficiency in equipment and infrastructure is flagrant and constitutes a barrier to the management of neuro-urological disorders.

Then again, the specific social care provided by the health insurance companies also requires strengthening. The family’s solidarity is predominant instead of being secondary.

The main ‘points of light’ that have to be encouraged are the efforts of the Faculty of Medicine in Oujda to establish an international university diploma in urodynamic explorations and particularly the far-reaching experience of the Mohammed V Military Hospital in Rabat in the field of neurostimulation.

But if we can discuss this subject in its entirety, we believe as urology professors, that the progress in Moroccan neuro-urology depends both on intrinsic factors of the mentioned subspecialty and also on extrinsic factors relating to the practice of urology in general in our country.

It should be noted that neuro-urology is a fairly young subspecialty that was initially a discipline submerged in other subspecialties under different terms: bladder dysfunction, low urinary tract symptoms, reconstructive surgery, functional urology, female urology, pelvic reconstruction. For instance, if we look at the history of the international continence society, the leading society in functional urology founded in 1971, it did not recognize neuro-urology as a subspecialty until 2004 [3]. While the term neuro-urology in guidelines was used by the European Association of Urology for the first time in 2014 [4]. Theoretical teaching and practical training in neuro-urology were considered insufficient by 73.9% and 64.2% respectively of urologists in training in France in 2010 [5].

On this basis, it seems that neuro-urology in Morocco will take time to organize and structure itself as a real subspecialty. This development will be affected by both the expertise of Moroccan urologists and the parallel evolution of social and paramedical organizations. In Morocco, continuing medical education rarely takes the form of a full university course, as it is essentially based on occasional training in the form of congresses, workshops, or university diplomas (which is inspired from the French system and based mainly on theoretical teaching). The occasional aspect, the absence of regular postgraduate medical knowledge evaluation, and the weakness of the practical side should encourage the adoption of other training models.

At the national level, Moroccan medical faculties offer seven university diplomas in urology and six in clinical research every academic year (Table 1). These courses are quantitatively insufficient, and their practical side remains weak or absent. Indeed, the international trend is currently towards fellowships, which are high-level super- training courses following a well-structured academic curriculum based on clinical research. In 2018, 50% of American urology residents undertook fellowships after their residency [6], while in Saudi Arabia in 2021, for example, this rate rises to 96.5% [7].

**Table 1.** Moroccan university diplomas of urology and research

Faculty of Medicine	Urology diploma	Research diploma
Fes		- Redaction Medicale Scientifique - Epidemiologie et Recherche Clinique - Methodes de Recherche en Cancerologie
Oujda	- Explorations Perinelaes et Urodynamique - Techniques de Base en Chirurgie Laparoscopique	
Rabat	- Onco-urologie - Coeliochirurgie	- Essais Cliniques - Biostatistique et Méthodologie de Recherche Clinique
Casablanca	- Onco-urologie - Chirurgie Laparoscopique - Sexothérapie Clinique	- Biostatistique et Mesure de la Santé Perceptuelle

A Neuro-urology Fellowship should include both theoretical and practical education, with clinical and research paths over a minimum period of 1 year and an evaluation at the end of the training. Such training can only enrich expertise and clinical research in all areas of urology.

In our opinion, the development of expert centers able to provide an advanced level of care can only be conceived from the perspective of subspecialised practice. A proliferation of medical universities and hospital centers, and an increase in the number of urologists will be the first step towards this objective.
